# The Role of Health-related Behaviors in Gestational Weight Gain among Women with Overweight and Obesity: A Cross-sectional Analysis

**DOI:** 10.1055/s-0040-1712132

**Published:** 2020-06

**Authors:** Daiane Sofia Morais Paulino, Maira Pinho-Pompeu, Fernanda Raikov, Juliana Vasconcellos Freitas-Jesus, Helymar Costa Machado, Fernanda Garanhani Surita

**Affiliations:** 1 Department of Obstetrics and Gynecology, Universidade Estadual de Campinas, Campinas, SP, Brazil

**Keywords:** health-related behaviors, gestational weight gain, overweight, obesity, comportamentos relacionados à saúde, ganho de peso gestacional, sobrepeso, obesidade

## Abstract

**Objective** To evaluate the influence of health-related behaviors including food intake, physical activity, sleep time, smoking habits, stress, depression, and optimism on excessive gestational weight gain (GWG) among women with overweight and obesity.

**Methods** A cross-sectional study was conducted at the Women's Hospital of the Universidade de Campinas, Campinas, state of São Paulo, Brazil, with 386 mediate postpartum women that fit the inclusion criteria of ≥ 19 years old, first prenatal care visit at or before 14 weeks, and single live baby. Dietary habits, physical exercise practice, sleep duration, smoking and alcohol habits were self-reported. Psychosocial history was evaluated using the Edinburgh Postpartum Depression Scale (EPDS), Perceived Stress Scale (PSS), and Life Orientation Test-Revised (LOT-R). Sociodemographic, obstetric, anthropometric, and neonatal data were retrieved from medical records. Descriptive statistics and stepwise logistic regression were performed.

**Results** The prevalence of overweight and obesity was 29.27% and 24.61%, respectively, according to the body mass index (BMI). Excessive GWG was observed in 47.79% of women with overweight and in 45.26% of women with obesity. Excessive GWG among overweight and obese women was associated with inadequate vegetable and bean consumption (odds ratio [OR] = 2.95, 95% confidence interval [CI]: 1.35–6.46 and OR = 1.91; 95%CI: 1.01–3.63, respectively) and stress (OR = 1.63; 95%CI 1.01–2.64). After adjustment by maternal age, multiparity, sleep duration, smoking, and alcohol intake, we found that stress (PSS ≥ 20) was associated with excessive GWG in women with overweight or obesity (OR: 1.75; 95%CI: 1.03–2.96).

**Conclusion** Among women with overweight and obesity, stress is the main variable associated with excessive GWG. Inadequate vegetables and beans consumption also showed association with excessive GWG.

## Resumo

**Objetivo** Avaliar a influência de comportamentos relacionados à saúde: ingestão alimentar, atividade física, tempo de sono, tabagismo, estresse, depressão e otimismo no ganho de peso gestacional (GPG) excessivo em mulheres com sobrepeso e obesidade.

**Métodos** Estudo transversal no Hospital da Mulher, Universidade de Campinas, Campinas, SP, Brasil, com 386 mulheres no puerpério mediato, ≥ 19 anos, primeira consulta pré-natal até 14 semanas e cuja gestação resultou em neonato vivo. Os comportamentos relacionados à saúde foram autorreferidos. História psicossocial foi avaliada usando: Escala de Depressão Pós-Parto de Edimburgo (EPDS, na sigla em inglês), Escala de Estresse Percebido (PSS, na sigla em inglês) e Teste de Orientação à Vida-Revisado (LOT-R, na sigla em inglês). Dados sociodemográficos, obstétricos, antropométricos e neonatais foram obtidos dos prontuários médicos. Realizou-se análises descritivas e regressão logística.

**Resultados** A prevalência de sobrepeso e obesidade foi de 29,27% e de 24,61%, respectivamente. Ganho de peso gestacional excessivo foi observado em 47,79% das mulheres com sobrepeso e em 45,26% das mulheres com obesidade. O consumo inadequado de verduras e feijão (razão de probabilidade [OR] = 2,95; índice de confiança [IC] 95%: 1,35–6,46 e OR = 1,91; IC95%: 1,01–3,63, respectivamente) e estresse (OR = 1,63; IC95%: 1,01–2,64) foram associados ao GPG excessivo em mulheres com sobrepeso e obesidade. Análises ajustadas para idade materna, multiparidade, duração do sono, tabagismo e ingestão de álcool mostraram que o estresse (PSS ≥ 20) associou-se ao GPG excessivo em mulheres com sobrepeso e obesidade (OR = 1.75; 95%CI: 1.03–2.96).

**Conclusão** Entre mulheres com sobrepeso e obesidade, o estresse foi a principal variável associada ao GPG excessivo. O consumo inadequado de verduras e feijão também se associou com o GPG excessivo.

## Introduction

Obesity and overweight prevalence have increased dramatically over the past 30 years among reproductive-age women.^1^ Obesity in the gestational period has consistently been associated with adverse health outcomes for mothers and their children, including epigenetic alterations and metabolic disorders in offspring.^2^

Gestational weight gain (GWG) is an important modulator of perinatal outcomes that is independent of prepregnancy nutritional status.^3^ In this way, it is described that excessive GWG increases the risk of gestational diabetes mellitus, hypertensive syndrome, and cesarean section (C-section).^4^ Besides that, excessive GWG has been correlated with postpartum weight retention, obesity, and future metabolic disorders in women.^4^ In the children, excessive GWG is associated with macrosomia and increased adiposity in infants, whereas insufficient GWG is associated with lower birthweight and preterm birth.^3^ ^4^

Despite the adverse effects of excessive GWG, the influence of health-related behaviors on GWG is not completely understood. The association between modifiable behavioral factors such as dietary habits, physical activity, and psychological states with excessive GWG is controversial.^5^ ^6^ ^7^

In this sense, a systematic review about the effectiveness of diet or exercise, or both interventions for preventing excessive GWG demonstrated that diet or exercise reduced the frequency of excessive GWG by 20% (relative risk [RR] = 0.8; 95% confidence interval [CI]: 0.73–0.87) while minimally increasing the risk for inadequate GWG (RR = 1.14; 95%CI: 1.02–1.27).^8^

The association between short sleep duration (SSD) and obesity is well-described, and the negative influence of SSD on energy balance regulation and its potential to impact on weight status has also been established.^9^ ^10^ Furthermore, during pregnancy, the prevalence of poor sleep quality was higher among overweight and obese women.^11^

Similarly, alcohol consumption and smoking are also correlated with energy metabolism. It was demonstrated that smoking cessation was associated with greater GWG.^12^ However, in animals, tobacco and alcohol exposure reduced GWG.^13^

In relation to the psychological factors, it was demonstrated that, among women with obesity and adequate GWG, emotional conflicts arising from fear of their own and their baby's life due to the inherent risks of obesity in this life cycle appear to be a potentiating factor for changing habits.^14^

Therefore, understanding the behaviors that influence GWG among women with overweight and obesity is crucial to developing behavioral change strategies and specific guidelines for weight control and prenatal education. Pregnancy is also an important period that affects maternal weight trajectory and fetal metabolic programming. The aim of the present study was to evaluate the effects of health-related behaviors (diet, physical activity, sleep duration, smoking, alcohol intake, and psychological factors) on excessive GWG among women that were overweight or obese.

## Methods

A cross-sectional study was conducted at the Women's Hospital of the Universidade de Campinas, Campinas, state of São Paulo, Brazil. The eligibility criteria were ≥ 19 years old, antenatal care starting in or before 14 weeks of gestation, and a pregnancy that resulted in a live singleton birth. The present study was approved by the Ethics Committee of the Universidade de Campinas and the Brazilian National Board of Health, CAAE 44630615.3.0000.5404. Data were collected after informed consent was obtained from all participants.

The required sample size was calculated based on the associations between modifiable health-related behaviors and excessive or insufficient GWG reported by McDonald et al.^15^ Assuming a significance level of 5% and power of 90%, the necessary sample size was 386 women.

Sociodemographic characteristics (maternal age and education level), obstetric history, anthropometric data, and neonatal data were collected from medical records. Education level was classified as elementary school (up to 9 years of study); high school (up to 12 years of study); college, university or advanced degree (> 12 years of study). The body mass index (BMI) early in pregnancy was calculated from height and weight at the first prenatal visit (up to 14 weeks) and classified according to the criteria of the World Health Organization (WHO). The final weight was defined as the weight at admission for delivery. Gestational weight gain was calculated as the weight difference between final weight and weight at the first prenatal visit, and was evaluated according to the guidelines of the Institute of Medicine.^16^ The adequacy of birthweight was classified according to the INTERGROWTH-21st Project Study^17^ recommendations, which consider the gestational age and gender and categorize the adequacy of birthweight as small for gestational age birthweight (SGA), adequate for gestational age birthweight (AGA) or large for gestational age birthweight (LGA).

The variables related to the adoption of health-related behaviors during pregnancy were collected in the mediate postpartum (1–3 days after delivery). Physical exercise practice, sleep duration, alcohol intake and smoking habits during the pregnancy were captured using a specially designed self-report questionnaire and the women referred about their habits during the entire gestational period.

Dietary habits were assessed once by a qualitative frequency questionnaire,^18^ which provides data on maternal food intake patterns based on healthy and unhealthy eating markers. The use of eating markers during pregnancy is recommended by the Brazilian Ministry of Health.^19^ Women were questioned regarding the habitual consumption frequency of foods that are recognized as healthy (fruits, vegetables, beans, and milk) and unhealthy (soft drinks, meat, and family oil consumption) eating markers. The intake of these foods was obtained as never or less than once/week; 1–3 times/week; 3–6 times/week or every day. The variable “family oil consumption” was obtained as numbers of tins of oil consumed by the family during a month.

Psychosocial issues were evaluated using a 10-item version of the Edinburgh Postpartum Depression Scale (EPDS),^20^ a 10-item version of the Perceived Stress Scale (PSS),^21^ and the Life Orientation Test-Revised (LOT-R).^22^ The EPDS is a depression screening tool in which scores ≥ 10 indicate possible depression. Perceived Stress Scale responses were scored using a 5-point scale (coded 0–4) and summed; scores ≥ 20 indicated a high level of perceived stress. The LOT-R consists of 6 items, and individual items scores are summed to yield a total score (range 6–30); high scores indicated optimism and low scores indicated pessimism. We treated the LOT-R score as a continuous variable. Moreover, we followed the time frame suggested in each instrument, which is “last 7 days” for the EPDS, “last month” for the PSS, and “usually” for the LOT-R.

## Data Analysis

Categorical variables were described using frequencies and percentages, whereas continuous variables were described using mean and standard deviation (SD). Logistic regression analysis was used to identify independent factors associated with excessive GWG considering health-related behaviors, as well as sociodemographic and obstetric variables in pregnant women with obesity and overweight compared with all other women in the sample (i.e., pregnant women with obesity and overweight with adequate and insufficient GWG plus eutrophic and underweight pregnant women with any GWG category). We also conducted a univariate and multivariate analysis (stepwise) adjusted for maternal age, multiparity, sleep hours, smoking status, and alcohol intake, considering that these factors are related to energy metabolism. The significance level for all tests was 5%, and they were performed using SAS Statistical Analysis System for Windows, version 9.2 (SAS Institute Inc., Cary, NC, USA). All items on the STROBE checklist for a cross-sectional study have been included in the present manuscript.

## Results

The sample comprised 386 women with a mean age of 28.31 years old (SD = 5.68) and a mean BMI of 27.98 kg/m^2^ (SD = 3.66). The combined prevalence of overweight and obesity was 53.88%. Obstetric complications were present in 47.13% of participants, with gestational diabetes mellitus (15.36%), hypertensive disorder (13.28%), and/or urinary infection (10.41%) occurring most frequently. Table 1 displays the BMIs as well as sociodemographic, obstetric, and neonatal variables of the participants.

**Table 1:** Socio-demographic and obstetric characteristics, prepregnancy body mass index, and perinatal outcomes for the whole sample

Variable (*n* = 386)	Mean	SD
**Maternal age (years old)**	**28.41**	**±0.28**
**BMI at first prenatal visit (kg/m^2^)**	**27.98**	**±3.66**
	***n***	**%**
Educational level*		
Elementary school	85	23.48
High school	215	59.39
College, university, or advanced degree	62	17.12
Number of pregnancies		
1	131	33.93
≥ 2	255	66.06
Delivery Mode		
C-section	177	45.85
Vaginal	209	54.14
BMI at first prenatal visit (kg/m^2^)		
< 18.5	16	4.15
18.5 a 24.9	162	41.97
25 a 29.9	113	29.27
≥ 30	95	24.61
Pre-pregnancy NCD	95	24.61
Obstetric complications† (384)	181	47.13
Diabetes mellitus	59	15.36
Hypertensive disorder	51	13.28
Urinary infection	40	10.41
Other	31	8.07
Adequacy of birthweight£		
SGA	34	8.81
AGA	297	76.94
LGA	55	14.25
Apgar in the fifth minute		
< 7	1	0.26
≥ 7	385	99.74

Abbreviations: AGA: Adequate-for-gestational-age; BMI: Body mass index, LGA: large-for-gestational-age; NCD: non-communicable disease. SGA: small-for-gestational-age.

† 2 missing data.

* 4 missing data.

£ 9 missing data. Data were presented as frequencies (n) and percentages (%).

**Fig. 1:**
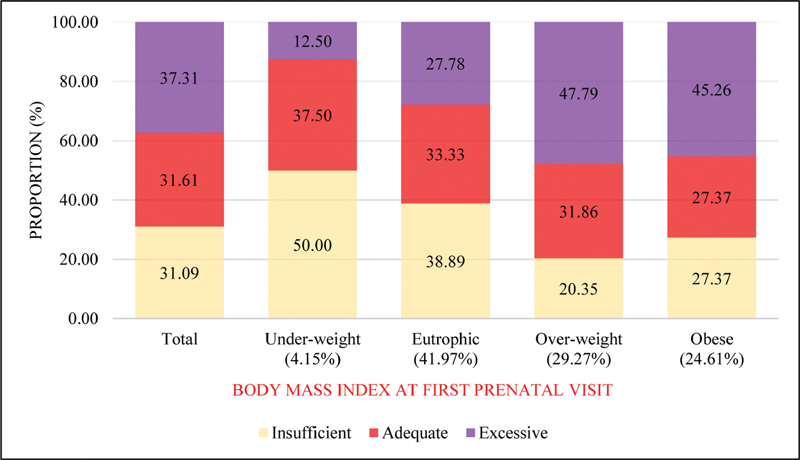
Prevalence of insufficient, adequate and excessive gestational weight gain by maternal body mass index at first prenatal visit (≤ 14 weeks).

Gestational weight gain was insufficient for 31.09%, adequate for 31.61%, and excessive for 37.31%. Excessive GWG among overweight and obese women was 47.79% and 45.26% respectively. Fig. 1 shows GWG adequacy according to maternal prepregnancy BMI.

Approximately 48% of the participants reported daily fruit and vegetable intake, 44.24% reported daily beans intake, and 63.73% reported daily intake of milk and dairy products. The majority of women (64.94%) reported daily consumption of meat.

More than half of the sample (57.03%) reported not practicing physical activity during pregnancy. Most women (91.91%) did not drink any alcoholic beverages during pregnancy. A total of 64 women (16.8%) reported some tobacco use during pregnancy, while 24 of these women (6.2%) reported smoking throughout pregnancy.

Sleeping < 7 hours per night before and during early pregnancy was reported by 38.12% and 32.90% of participants, respectively. Most women (59.37%) also reported sleeping < 7 hours during the final trimester of pregnancy.

Less than half of the women (40.68%) scored > 10 on the EPDS, 35.29% had PSS scores indicative of high perceived stress (PSS ≥ 20), and the mean LOT-R score was 15.67 ± 3.22. A detailed sample distribution for the health-related behaviors is presented in Table 2.

**Table 2:** Percentage distribution of health-related behaviors in the whole sample

Variable (n)	N (386)	%	Variable (n)	N (386)	%
Sleep time			Food Intake (days/week)		
Sleep Hours < 7			Vegetable intake (383)		
Before pregnancy (383)	146	38.12	7	187	48.83
Beginning of pregnancy (383)	126	32.90	3–6	62	16.19
End of pregnancy (379)	225	59.37	1–3	102	26.63
			Never or < 1	32	8.36
Physical Activity			Fruit Intake (386)		
Sedentary lifestyle (379)	215	57.03	7 (ref.)	186	48.19
			3–6	69	17.88
Unhealthy behaviors			1–3	99	25.65
Alcohol intake (383)	31	8.09	Never or < 1	32	8.29
Smoking- last 2 years (381)	64	16.80	Milk/dairy products intake (386)		
			7	246	63.73
Psychosocial History			3–6	41	10.62
EPDS (381)			1–3	61	15.80
< 10	226	59.32	Never or < 1	38	9.84
≥ 10	155	40.68	Meat intake (385)		
			Never or < 1	15	3.9
PSS (374)			1–3	58	15.06
< 20	242	64.71	3–6	62	16.10
≥ 20	132	35.29	7 (ref.)	250	64.94
			Soft drink intake (386)		
LOT-R (376) Mean ± SD	15.67	±3.22	Never or < 1	137	35.49
Food Intake (number of tins)			1–3	105	27.20
Family oil consumption (379)			3–6	60	15.54
< 1	108	28.50	7	84	21.76
1	108	28.50	Beans intake (382)		
2– 3	128	33.77	7 (ref.)	169	44.24
> 3	35	9.23	3–6	48	12.56
			1–3	106	27.74
			Never or < 1	59	15.44

Abbreviations: EPDS: Edinburgh Postpartum Depression Scale. PSS: Perceived Stress Scale. LOT-R: Revised Life Orientation Test.

One tin = 900ml. Data were presented as frequencies (n) and percentages (%).

Logistic regression analysis was conducted to evaluate the risk factors for excessive GWG among women with overweight and obesity when compared with those with adequate and insufficient GWG. The results are described in Table 3.

**Table 3:** Unadjusted risk factors (sociodemographic, obstetric and health-related behavior factors) for excessive gestational weight gain in women with overweight and obesity

Variable	OR	95%CI	Variable	OR		95%CI	Variable	O.R.	95% CI
			Meat intake (days/week)				Family consumption of oil (number of tins)		
Maternal Age (years old)	1.01	0.97–1.05	Never or < 1	0.73		0.20–2.66	< 1 (ref.)	1.00	—
Multiparity	1.13	0.69–1.84	1–3	1.01		0.53–1.95	1	1.54	0.81–2.95
			3–6	0.93		0.49–1.77	2–3	1.59	0.85–2.97
Pre-pregnancy NCD	1.25	0.75–2.11	7 (ref.)	1.00		—	> 3	2.30	0.98–5.37
			Beans				Vegetable intake (days/ week)		
Smoking in the last 2 years	0.89	0.48–1.68	7 (ref.)	1.00		—	7 (ref.)	1.00	—
Sedentary lifestyle	1.23	0.78–1.95	3–6	0.80		0.35–1.79	3–6	1.21	0.61–2.39
Alcohol intake during pregnancy	0.30	0.09–1.01	1–3	1.30		0.74–2.27	1–3	1.51	0.86–2.63
			Never or < 1	1.91		1.01–3.63	Never or < 1	2.95	1.35–6.46
Educational level			Fruit Intake (days/week)			—	Sleep Hours < 7		
Elementary school	1.08	0.61–1.91	7 (ref.)	1.00		0.61–2.21	Before pregnancy	0.93	0.58–1.50
High school (ref.)	1.00	—	3–6	1.16		0.89–2.67	Beginning of pregnancy	0.90	0.55–1.49
Advanced degree	0.93	0.48–1.79	1–3	1.54		0.59–3.22	Final of pregnancy	0.94	0.59–1.51
			Never or < 1	1.38					
Soft drink intake (days/week)			Milk and dairy products intake (days/ week)				LOT-R	0.97	0.90–1.04
Never (ref.)	1.00	—	7 (ref.)	1.00		—	EPDS		
< 1	2.05	0.81–5.18	3–6	0.88		0.41–1.89	< 10 (ref.)	1.00	—
1–3	2.02	0.81–5.03	1–3	0.89		0.47–1.70			
3–6	1.32	0.47–3.69	Never or <1	0.51		0.21–1.28	≥ 10	1.18	0.74–1.89
7	1.99	0.78–5.10					PSS		—
							< 20 (ref.) ≥20	1.63	1.01–2.64

Abbreviations: CI, confidence interval; EPDS, Edinburgh Portpartum Depression.Scale; LOT-R, Revised Life Orientation Test; NCD, noncommunicable disease; OR, odds ratio; PSS, Perceived Stress Scale; Ref, reference level.

Excessive gestational weight gain (GWG) in overweight and obese women (*n* = 97), remaining sample (*n* = 289). One tin = 900ml. Data were analyzed by logistic regression analyses.

We also performed univariate and multivariate logistic regression analysis to assess the risk factors for excessive GWG among women with overweight and obesity, after adjustment for maternal age, multiparity, sleep duration, smoking and alcohol intake (Table 4 and Table 5). Univariate logistic regression analysis showed that inadequate consumption of vegetables and beans (no intake or intake less than once per week) and stress were associated with excessive GWG among women with overweight and obesity.

**Table 4:** Adjusted risk factors for excessive gestational weight gain in women with overweight and obesity

Variable	OR	95%CI	Variable	OR	95%CI
Socio-demographic and antecedents			Food Intake (days/week)		
Educational level			Vegetable intake		
Elementary school	1.07	0.58–1.99	7	1.00	—
High school (ref.)	1.00	—	3–6	1.20	0.58–2.45
Advanced degree	0.83	0.48–1.68	1–3	1.54	0.86–2.77
Pre-pregnancy NCD			Never or < 1	2.81	1.24–6.36
			Fruit Intake		
No	1.00	—	7 (ref.)	1.00	—
Yes	1.13	0.65–1.94	3–6	1.19	0.61–2.33
Physical Activity			1–3	1.54	0.87–2.73
			Never or < 1	1.32	0.54–3.23
Sedentary lifestyle	1.21	0.75–1.97	Milk/dairy products intake		
Psychosocial History			7	1.00	—
			3–6	0.88	0.40–1.90
EPDS			1–3	0.77	0.39–1.50
< 10	1.00	—	Never or < 1	0.54	0.21–1.37
≥ 10	1.16	0.71–1.88	Beans intake		
			7 (ref.)	1.00	—
PSS			3–6	0.84	0.37–1.90
< 20	1.00	—	1–3	1.41	0.79–2.51
≥ 20	1.76	1.07–2.90	Never or < 1	2.02	1.03–3.99
			Meat intake		
LOT-R	0.97	0.90–1.05	Never or < 1	0.70	0.18–2.63
Food Intake (number of tins)			1–3	1.21	0.61–2.38
			3–6	0.93	0.48–1.81
Family oil consumption			7 (ref.)	1.00	—
< 1	1.00	—	Soft drink intake		
1	1.54	0.80–2.98	Never or < 1	1.00	—
2– 3	1.56	0.82–2.95	1–3	2.17	0.85–5.57
> 3	2.31	0.97–5.53	3–6	1.38	0.48–3.94
			7	2.37	0.89–6.31

Abbreviations: CI, confidence interval; EPDS, Edinburgh Portpartum Depression.Scale; LOT-R, Revised Life Orientation Test; NCD, noncommunicable disease; OR, odds ratio; Ref, reference level.

Excessive gestational weight gain (GWG) in overweight and obese women (*n* = 94), remaining sample (*n* = 277). One tin = 900ml. Data were analyzed by logistic regression analyses adjusted by maternal age, multiparity, sleep duration, smoking and alcohol intake.

**Table 5:** Multivariate logistic regression analysis between adjusted risk factors and excessive gestational weight gain in women with overweight and obesity

Variable	*p-value*	OR	95%CI
PSS (Stress)			
< 20 (ref.)	—	1.00	—
≥ 20	0.037	1.75	1.03–2.96

Abbreviations: OR, Odds Ratio; CI, confidence interval; PSS: Perceived Stress Scale; Ref, reference level.

Excessive gestational weight gain (GWG) in overweight and obese women (*n* = 83), remaining sample (*n* = 249).. Multivariate logistic regression adjusted by maternal age, multiparity, sleep duration, smoking and alcohol intake. Independent variables considered: pre-pregnancy non-communicable disease (no: 0, yes: 1); physical activity (no: 1, yes: 0); maternal education level (elementary: 1, high school: 0, advanced degree: 2); soft drink intake (never or < 1 day/week: 0, 1–3 days/week: 1, 3–6 days/week: 2, 7 days/week: 3); meat intake (never or < 1 day/week: 1, 1–3 days/week: 2, 3–6 days/week: 3, 7 days/week: 0); beans intake (never or < 1 day/week: 1, 1–3 days/week: 2, 3–6 days/week: 3, 7 days/week: 0); fruit intake (never or < 1 day/week: 1, 1–3 days/week: 2, 3–6 days/week: 3, 7 days/week: 0); milk intake (never or < 1 day/week: 1, 1–3 days/week: 2, 3–6 days/week: 3, 7 days/week: 0); vegetable intake (never or < 1 day/week: 1, 1–3 days/week: 2, 3–6 days/week: 3, 7 days/week: 0); family oil consumption (< 1 tin: 0, 1 tin: 1, 2–3 tin: 2, > 3: 3); LOTR (continuous variable), EPDS (< 10: 0, ≥ 10: 1) and PSS (< 20: 0, ≥ 20: 1).

Multivariate stepwise logistic regression adjusted by maternal age, multiparity, sleep duration, smoking, and alcohol intake demonstrates that stress (PSS ≥ 20) approximately doubles the likelihood of excessive GWG in women with overweight or obesity (OR = 1.75; 95%CI: 1.03–2.96) (Table 5).

## Discussion

The present study identified that stress is the main variable associated with excessive GWG in women that were obese or overweight. Low vegetable and beans consumption during pregnancy (no intake or intake less than once per week) also showed a strong association.

In relation to stress due to psychological factors, one study recently demonstrated that lower reported stress was associated with a greater likelihood of pregnant women achieving adequate GWG.^23^ In contrast to these findings, another study found no association between stress and excessive GWG.^24^ Race/ethnicity and socioeconomic status were described as determinants of maternal stress, while parental stress negatively affected quality of life among women.^25^

The association between dietary patterns and diet quality with GWG has been previously described;^26^ ^27^ however, the results remain controversial. While one study demonstrated that women who consumed at least three servings of fruits and vegetables per day presented less weight gain,^27^ another study found no relationship between vegetable and fruit consumption and GWG.^28^

The majority of our sample reported not eating beans every day. This result is in agreement with the last Household Budget Survey that indicated the decreased consumption of foods considered healthy and popular in Brazilian culture, such as beans, fruits, and vegetables.^29^ In the habitual Brazilian diet, beans and meat are responsible for the majority of iron intake, which is described as the principal immediate factor to anemia.^30^ Moreover, a diet pattern rich in fish, beans, nuts, and yogurt has been shown to decrease the risk of inadequate GWG.^31^

Most women in the present study were overweight or obese, and consistent with other studies,^32^ ^33^ the highest prevalence of excessive GWG occurred among women that were overweight or obese.

We did not determine that SSD was associated with excessive GWG in women with overweight and obesity. In contrast, one study that objectively assessed sleep duration demonstrated that excessive GWG was associated with SSD and more sleep disruption among prepregnancy overweight women.^34^

Notably, some study limitations should be considered upon interpreting our results. First, our study is limited by the use of non-quantitative assessment of dietary intake, which precludes the estimation of caloric, macronutrient, and micronutrient intake. Second, sleep duration was self-reported rather than measured, and sleep quality is unknown. Lastly, our study has a cross-sectional design. As a result, the simultaneous exposure and outcome assessment does not allow for the establishment of a temporal relationship between exposure and outcome.

In summary, our findings highlight that some dietary patterns during pregnancy (e.g., low vegetable and bean intake) and stress may contribute to excessive GWG in overweight and obese women. Although these findings should be confirmed by further studies, pregnancy is a powerful impetus for positive behavioral change. Based on our results, a multidisciplinary approach — especially with nutritional attention and psychologist support — should be used for overweight and obese women to promote adequate GWG and optimize antenatal care.

## Conclusion

Among women with overweight and obesity, health-related behavior stress is the main variable associated with excessive GWG. Low vegetable and beans consumption during pregnancy (no intake or intake less than once per week) also showed strong association with excessive GWG. The current findings provide information that can be used to design intervention programs for women with overweight and obesity in early pregnancy to improve maternal and child health on the basis that pregnancy is an ideal window of opportunity for women to adopt healthier lifestyles. Further studies, with a larger and multicenter cohort, should be conducted to confirm these data.
